# Pri smORF Peptides Are Wide Mediators of Ecdysone Signaling, Contributing to Shape Spatiotemporal Responses

**DOI:** 10.3389/fgene.2021.714152

**Published:** 2021-08-30

**Authors:** Azza Dib, Jennifer Zanet, Alexandra Mancheno-Ferris, Maylis Gallois, Damien Markus, Philippe Valenti, Simon Marques-Prieto, Serge Plaza, Yuji Kageyama, Hélène Chanut-Delalande, François Payre

**Affiliations:** ^1^Molecular, Cellular and Developmental Biology Department (MCD), Centre de Biologie Intégrative (CBI), CNRS, UPS, University of Toulouse, Toulouse, France; ^2^Department of Biology, Graduate School of Science, Kobe University, Kobe, Japan; ^3^Biosignal Research Center, Kobe University, Kobe, Japan

**Keywords:** smORF peptides, polished rice, tarsal less, mille pattes, ecdysone, steroids

## Abstract

There is growing evidence that peptides encoded by small open-reading frames (sORF or smORF) can fulfill various cellular functions and define a novel class regulatory molecules. To which extend transcripts encoding only smORF peptides compare with canonical protein-coding genes, yet remain poorly understood. In particular, little is known on whether and how smORF-encoding RNAs might need tightly regulated expression within a given tissue, at a given time during development. We addressed these questions through the analysis of *Drosophila polished rice* (*pri*, a.k.a. *tarsal less* or *mille pattes*), which encodes four smORF peptides (11–32 amino acids in length) required at several stages of development. Previous work has shown that the expression of *pri* during epidermal development is regulated in the response to ecdysone, the major steroid hormone in insects. Here, we show that *pri* transcription is strongly upregulated by ecdysone across a large panel of cell types, suggesting that *pri* is a core component of ecdysone response. Although *pri* is produced as an intron-less short transcript (1.5 kb), genetic assays reveal that the developmental functions of *pri* require an unexpectedly large array of enhancers (spanning over 50 kb), driving a variety of spatiotemporal patterns of *pri* expression across developing tissues. Furthermore, we found that separate *pri* enhancers are directly activated by the ecdysone nuclear receptor (EcR) and display distinct regulatory modes between developmental tissues and/or stages. Alike major developmental genes, the expression of *pri* in a given tissue often involves several enhancers driving apparently redundant (or shadow) expression, while individual *pri* enhancers can harbor pleiotropic functions across tissues. Taken together, these data reveal the broad role of Pri smORF peptides in ecdysone signaling and show that the *cis-*regulatory architecture of the *pri* gene contributes to shape distinct spatial and temporal patterns of ecdysone response throughout development.

## Introduction

Recent advances in functional genomics indicate that apparently non-coding RNAs often encode smORF peptides (Andrews and Rothnagel, 2014; Pauli et al., 2015; Pueyo et al., 2016) and *Polished rice (pri)* represents a paradigm of this emerging field (Andrews and Rothnagel, 2014; Zanet et al., 2016). *Pri*, also known as *tarsal less* or *mille pattes*, was initially identified as a long non-coding RNA that exhibits highly dynamic expression during embryogenesis (Inagaki et al., 2005; Tupy et al., 2005). Further work has demonstrated that *pri* acts through the production of evolutionarily conserved peptides, encoded by smORFs. In *Drosophila*, *pri* encodes four smORF peptides (from 11 to 32 aa), all bearing the conserved LDPTGQY motif. Across insect species, *pri* is involved in multiple developmental processes and plays key roles in the production and patterning of posterior structures (Savard et al., 2006; Galindo et al., 2007; Kondo et al., 2007; Ray et al., 2019). Partial loss-of-function mutations have revealed the role of *pri/tarsal less* in the formation of adult appendages, mutant animals displaying atrophic legs with missing/fused distal segments (Galindo et al., 2007; Pueyo and Couso, 2008; Ray et al., 2019). The complete loss of *pri* function yet results in embryonic lethality; *pri* mutants display severe defects of the tracheal respiratory system (Kondo et al., 2007, 2010; Ozturk-Colak et al., 2016). Another prominent phenotype of *pri* embryos is a deep alteration of epidermal development, including the absence of trichomes (Galindo et al., 2007; Kondo et al., 2007). The insect body is decorated by a stereotyped pattern of epidermal cell extensions, called denticles or hairs, and collectively referred to as trichomes (Payre, 2004). Previous work has established the pivotal role of a transcription factor, Ovo/Shavenbaby (Svb), whose expression defines the trichome pattern (Payre et al., 1999; Delon et al., 2003; Stern and Frankel, 2013; Crocker et al., 2015). Svb induces the expression of cellular effectors that reorganize the cytoskeleton (Menoret et al., 2013), extracellular matrix (Fernandes et al., 2010), as well as cuticle composition (Andrew and Baker, 2008) or pigmentation (Chanut-Delalande et al., 2006, 2012). Pri peptides are essential for trichome formation, since they induce a posttranslational conversion of Svb from a large-sized repressor to a shorter activator, which is required to trigger the expression of trichome effectors (Kondo et al., 2010). Pri peptides act through their binding to an E3 ubiquitin-ligase, called Ubr3, which allows the recruitment of the Ubcd6/Ubr3 complex on the Svb N-terminal region. Ubiquitination of the Svb N-terminal region leads to limited proteasome degradation of the Svb repressor domain, ultimately releasing the processed Svb activator protein (Zanet et al., 2015). Thus, the program of trichome differentiation is set up with the accumulation of the Svb repressor and kept on hold until *pri* is expressed.

**FIGURE 1:**
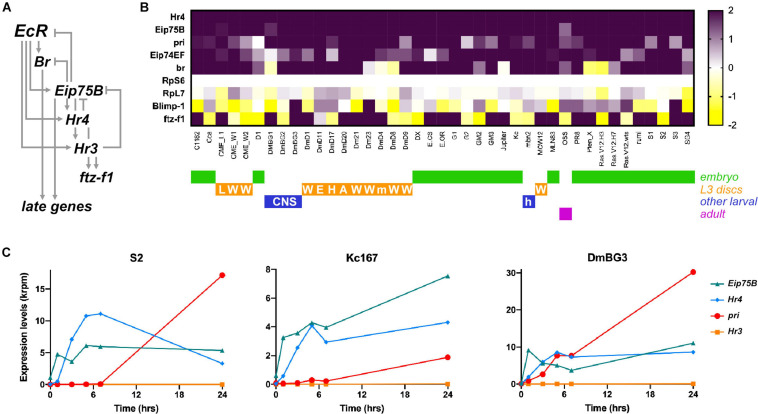
*pri* expression is strongly induced in the response to ecdysone in *Drosophila* cell lines. **(A)** Scheme of the canonical temporal cascade of TFs induced by ecdysone at metamorphosis onset [adapted from Ou and King-Jones (2013)]. Upon activation by ecdysone, EcR activates the expression of early-acting TFs (Br, Eip75B), then EcR needs the activity of early TFs to trigger the expression of additional genes (Hr4, Hr3) mediating the response to ecdysone. **(B)** Heat map showing differential expression of genes (Log2FC) encoding ecdysone-response TFs (Hr4, Eip75B, Eip74EF, Br, Blimp-1, or ftz-f1) and *pri* in 41 *Drosophila* cell lines after *vs.* before 5 h of ecdysone treatment. RpS6 and RpL7 are used as control genes. Raw data from Ref. (Stoiber et al., 2016) were reprocessed to analyze *pri* expression, as well as of all *Drosophila* genes to allow direct comparison. The origin of every cell line is color coded. L, leg disk; W, wing disk; E, eye/antennal disk; H, haltere disk; m, mixed imaginal disks; A, antennal disk; h, hemolymph; CNS, central nervous system. **(C)** Time course expression of *Eip75B*, *Hr4*, *pri*, and *Hr3* after ecdysone addition in cultured S2, Kc167, and DmBG3 cells. Raw data are from Ref. (Stoiber et al., 2016).

Accumulated evidences support that the developmental function of this sophisticated mechanism of *pri* action on Svb activity is to provide a strict temporal control of epidermal differentiation, in response to the systemic ecdysone hormone. Indeed, epidermal cells need to secrete a new cuticle exoskeleton before each molt (Moussian, 2010) and during the ultimate round of epidermal differentiation that occurs during metamorphosis for adult epidermal derivatives. Periodic pulses of ecdysone, the main steroid hormone in insects, provide timing cues well-known to trigger larval molting and metamorphosis (Yamanaka et al., 2013). Ecdysone is synthetized from dietary cholesterol *via* a series of enzymatic reactions leading to 20-hydroxyecdysone (20E) (Petryk et al., 2003), which binds to, and activates, the nuclear ecdysone receptor (EcR), often dimerizing with another nuclear receptor ultraspiracle (Usp) (Yao et al., 1992; Hall and Thummel, 1998; Ghbeish et al., 2001). The mechanisms of the response to ecdysone have been extensively studied at the onset of metamorphosis, which relies on a temporal series of transcriptional regulation (Thummel, 2001; Ou and King-Jones, 2013; Cheatle Jarvela and Pick, 2017). In the first step, 20E-bound EcR directly activates the expression of early genes that comprise Eip75B and broad-complex (Br) TFs (Thummel, 2001; Ou and King-Jones, 2013). Then, 20E/EcR acts together with early TFs to trigger the expression of additional tiers of early–late TFs, e.g., Hr4 and Hr3, activating, in turn, the expression of late genes (which are no longer under direct CONTROL OF EcR); inhibitory feedback loops ensuring tight temporal regulation (see Figure 1A). Ecdysone is also critical during embryonic development (Bender et al., 1997; Kozlova and Thummel, 2003), and EcR-responsive TFs are expressed in late embryos, following a similar temporal cascade (Ruaud et al., 2010). Consistently, we found that *pri* epidermal expression during both embryogenesis and metamorphosis is activated in response to ecdysone (Chanut-Delalande et al., 2014). Recent studies further show that *pri* expression is also controlled by ecdysone in the embryonic tracheal cells (Taira et al., 2021), as well as in the adult stem cells that regenerate the gut (Al Hayek et al., 2021) and kidneys (Bohère et al., 2018) to ensure homeostasis of vital functions across adulthood.

Here we show that Pri peptides further act as widespread mediators of ecdysone signaling across embryonic and post-embryonic development. Analysis of genome-wide transcriptional response to ecdysone reveals that the induction of *pri* expression is a hallmark of most cell types, when assayed *ex vivo*. *In vivo*, *pri* displays specific patterns of expression in various ecdysone-responsive tissues, during embryogenesis, larval/pupal stages, and in the adult. Genetic assays show that *pri* function relies on a large genomic region, which harbors multiple tissue- and stage-specific enhancers. Focusing on the embryonic epidermis and the adult leg primordium, we identified two *pri* enhancers whose activity is directly controlled by EcR, using distinct binding sites across tissues and between developmental stages.

## Materials and Methods

### Fly Stocks

Most mutants were obtained from the Bloomington Drosophila Stock Center^1^. The strains we used were *spo[1]/TM3,Dfd-YFP*, *phm[E7]/FM7a*, *Dfd-YFP*, *UAS-EcRDN* (Cherbas et al., 2003), *pri1 and pri3 mutants* (Kondo et al., 2007), *UAS-white-RNAi (BL28980)*, *UAS-EcR-RNAi* (BL#50712, BL#29374, BL58286), *Ptc-Gal4*, *en-Gal4*, *Dll-Gal4*, *UAS-pri* (Kondo et al., 2007), *UAS-mRFP* (BL#30567), and *tubGal80*^*ts*^ (BL#7017).

### DNA Constructs and Transgenic Lines

LacZ reporter constructs were produced by inserting 5- to 6-kb DNA fragments of the *pri* genomic region into the pAttB-LacZ (Menoret et al., 2013) vector. GFP reporters correspond to the same *pri* regulatory regions inserted into the pS3AG vector (gift from T. Williams). All constructs were verified by sequencing. Transgenic lines, including BAC constructs [from P(acman) Resources^2^], were generated using the PhiC31 system, and all inserted at the 86F position (BestGene). BAC transgenic lines were recombined with *pri*^1^ mutants. *w*^+^ candidate recombinants were screened for *pri*^1^ mutation by PCR using a primer located in the promoter region of *pri* and a primer in the residual P element present in the *pri*^1^ deficiency. Additional details are provided in Supplementary Tables 1, 2.

The function of EcR was depleted by expressing *UAS-EcRDN* driven by *ptc-Gal4* in the embryonic epidermis, and *UAS-EcRDN* driven by *dll-Gal4* and *UAS-EcRDN* or *UAS-EcR-RNAi* driven by *en-gal4*, *UAS-mRFP*; *tubGal80*^*ts*^ in larval/pupal legs.

### Analysis of ChIP-seq and RNA-seq Data

RNA-seq raw data (GSE11167) from Stoiber et al. (2016) were downloaded and reprocessed for bioinformatic and statistical analysis. Reads were mapped on the *Drosophila* genome (release r6.13) using STAR (Dobin et al., 2013) (v 2.5.2b, default parameters). Read count was done with HTseq-count (Anders et al., 2015) (v0.6.0, -t exon -r pos -i gene_symbol). We determined counts for *pri* with SAMtools (Li et al., 2009) (v 1.11), using values corresponding to the transcribed region of *pri* (samtools view “3R:13813109-13814648”). Statistical analyses were performed with edgeR (Robinson et al., 2010), using negative binomial generalized log linear model to the normalized (TMM) read count for each gene (gmLRT). Using R, we next calculated log_2_FC for either one sample for most cell lines, or by averaging values when two samples or more were available. Log_2_FC was considered when expression levels were ≥5 in control conditions. Heat map and plots were drawn using Prism 8.

### Embryo, Larval, and Pupal Staining

Embryos were dechorionated by bleach treatment, fixed in heptane saturated in paraformaldehyde for 20 min, devitellinized with heptane/methanol, and stored in methanol. Homozygous mutant embryos were identified by the absence of balancer chromosome (marked with GFP/YFP or LacZ). Sibling controls and mutant embryos were processed in the same batch; a typical collection includes >300 embryos in total. Staining was performed as previously described (Fernandes et al., 2010) using anti-ßgal (1/400), biotinylated goat anti-rabbit or anti-mouse (1/1,000, Vector Laboratories) preincubated with streptavidin-HRP (Vector Laboratories), and revelation was performed with DAB (3,3′-diaminobenzidine, Sigma). For phalloidin staining, embryos were treated with 80% ethanol instead of methanol to preserve actin organization. We used TRITC-phalloidin (Sigma) and AlexaFluor-488 (1/1,000, Molecular Probes) secondary antibodies. *In situ* hybridization to *pri* mRNA was processed using a DIG-labeled RNA antisense probe synthesized *in vitro* and reacted with alkaline phosphatase-conjugated anti-DIG antibody (Roche) as described previously (Chanut-Delalande et al., 2006).

For experiments with imaginal disks, expression of UAS constructs was induced at the L3 stage for 24 h at 29°C to inactivate *Gal80*^*ts*^. Imaginal disks from mid L3 larvae and pupae (between 0 and 4 h APF) were dissected in PBS and fixed in 4% paraformaldehyde/PBS at room temperature for 20 min. Tissues were rinsed three times in PBS for 10 min and permeabilized in PAT (1 × PBS, 0.3% Triton 100X, BSA 0.3%) for 1 h. For LacZ reporters, tissues were incubated with anti-βgal (1:1,000, Torrey Pines) overnight at 4°C in PAT, then rinsed in PAT and incubated for 2 h with Alexa-488 anti-rabbit secondary antibody (1:1,000, Jackson Immunoresearch). For GFP reporters, we directly observed the fluorescence of GFP. Nuclei were stained with TOPRO or DAPI, and samples were further dissected and mounted in Vectashield (Vectorlabs). Images were acquired with a SP8 Leica confocal microscope.

### Cuticle Preparation and Rescuing Assays

To assay the rescuing ability of BAC transgenes, *pri^3^/TM3-Dfd-LacZ* individuals were crossed by *pri*^1^,*BACxyz(mw+)/TM3-Dfd-LacZ* recombinants. To score for trichome formation, embryos were genotyped using X-Gal staining, and cuticles were prepared in Hoyers/lactic acid (1/1). Each rescuing experiment has been performed, independently, at least three times. To score for full rescue up to viable adults, the progeny of *pri^3^/TM3-Dfd-LacZ* X *pri^1^,BACxyz(mw+)/TM3-Dfd-LacZ* crosses was analyzed and the percentage of rescue estimated by the ratio between numbers of *w+* individuals (rescued) and *w+/TM3* siblings.

## Results

### *Pri* Is a Widespread Ecdysone Response Gene

To investigate the role of *pri* in the response to ecdysone, we first analyzed RNA-seq data profiling the transcriptional changes induced by ecdysone, across a broad set of cell lines from various origins (Stoiber et al., 2016). Each out of the 41 analyzed cell lines displays large-scale response to ecdysone, with several hundred of genes being differentially expressed upon ecdysone treatment (Stoiber et al., 2016). Unexpectedly, only a few genes exhibit general ecdysone response across cell lines, including *Hr4* and *Eip75B* that are activated in all cell lines, or *Eip74EF* and *broad* that are activated in most of them (Stoiber et al., 2016). *ftz-f1* displays more varying responses to ecdysone, while *Blimp-1* is often down regulated (Figure 1B).

However, the study of Stoiber et al. (2016) did not report the expression of *pri*, probably since this atypical polycistronic gene is often mis-annotated and/or not properly taken into account by standard bioinformatic pipelines. We, thus, reanalyzed raw data and mapped sequence reads that match the transcribed region of *pri* (3R:13,813,109..13,814,648) in every cell line, with or without ecdysone treatment. The results show that *pri* is generally not expressed, or with barely detectable levels, prior to ecdysone addition. In contrast, *pri* levels are robustly induced upon ecdysone treatment (log_2_FC > 1) in the vast majority (35 out of 41) of cell lines, derived from embryos, larval imaginal disks or central nervous system (Figure 1B). In most cases, the activation of *pri* expression was very strong (24 lines with log_2_FC > 2), *pri* belonging to the top five most induced genes in eight cell lines (induction > 85×), and showing the strongest upregulation in DmD4 cells (>800×).

In addition, Stoiber et al. (2016) performed an extended time course analysis of the response to ecdysone in three cell lines (S2, Kc, and BG3-c2). Consistent with the temporal cascade that occurs during the larval/pupal transition, the induction of *Eip75* is seen soon after ecdysone addition, while *Hr4* induction starts slightly later and peaks around 7–10 h of treatment. The expression of *pri* starts later and keeps increasing to reach very high levels, as seen 24 h after ecdysone exposure (Figure 1C). These data, thus, suggest that *pri* expression requires both 20E-bound EcR and other early TFs, as expected for early–late response genes (Ou and King-Jones, 2013). It is, thus, likely that 5 h of treatment (the conditions used in all other cell lines) is not optimal to capture the induction of *pri* by ecdysone, suggesting that longer exposure times might reveal *pri* response in even broader contexts.

Together, these data indicate that *pri* expression is induced by ecdysone in a large variety of cell types. They further suggest that *pri* is a broad target of ecdysone signaling, representing a novel member of the core response to steroids.

### *Pri* Function Relies on a Large Genomic Region

**FIGURE 2:**
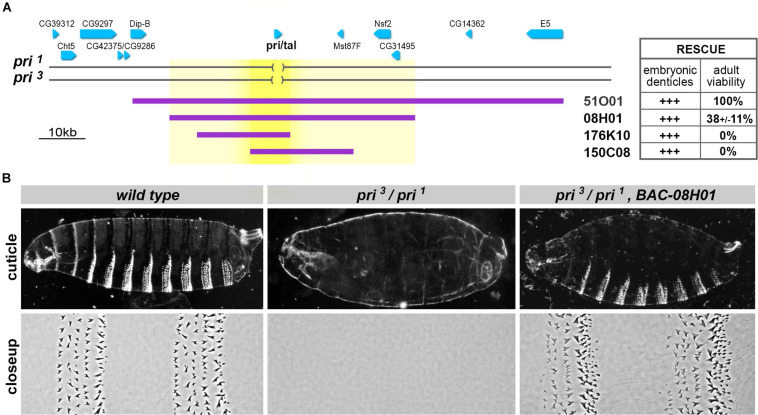
The *polished rice* functional unit extends over a large genomic region. **(A)** Schematic representation of the genomic region encompassing the *pri* locus. Genes are drawn as blue arrows that reflect the direction of transcription, and deleted regions in the *pri*^1^ and *pri*^3^ null alleles are indicated as brackets. Purple lines show the genomic regions carried by Bacterial Artificial Chromosome (BAC) transgenes; the table summarizes their respective activity for the rescue of embryonic trichomes and the observed percentage of emerging adults (51O01, 100% *n* = 197; 08H01, 38 ± 11% *n* = 201; 176K10, 0% *n* = 305; 150C08, 0% *n* = 126). **(B)** Cuticle preparations of whole embryos (top) and close ups of the ventral region of abdominal segments A3–A4 (bottom). The denticle belts featuring wild-type embryos are lacking in *pri^1^/pri^3^* mutant embryos. BAC-08H01 significantly restores trichome formation in this *pri* null background, although denticle belts appear not always complete. In this and all other figures, embryos are oriented with the anterior on the left, and the ventral region at the bottom of each picture.

To delineate the regulatory landscape driving *pri* transcription, we next sought to define the extent of the genomic locus required for its function throughout development. The *pri* gene is transcribed as an intron-less polyadenylated RNA of 1.5 kb in length and separated from neighboring genes by large upstream and downstream intergenic regions (Figure 2A). We generated a series of transgenic lines bearing overlapping genomic constructs [selected from libraries of Bacterial Artificial Chromosomes (BAC) (Venken et al., 2009)] and assayed their rescuing activity when reintroduced in a genetic background lacking *pri* function. We used a *trans* allelic combination of two null *pri* alleles (*pri^1^/pri^3^*) and tested whether BAC constructs could restore (i) differentiation of embryonic trichomes and (ii) full development up to viable and fertile adults. All BAC constructs were able to significantly restore the formation of trichomes (Figures 2A,B). This result suggested that the minimal genomic sequence required for *pri* function in epidermal trichome formation was included in the region shared by all rescue constructs, which is 8.7 kb in length (bright yellow, Figure 2A). However, none of the two smaller BAC constructs (176K10 and 150C8, of 20.4 and 21.9 kb, respectively) was sufficient to restore viability and the progression throughout later developmental stages. In contrast, the large 51O01 construct (98.7 kb) fully rescued the emergence of adults when introduced in a *pri* null genetic context. A significant yet weaker rescuing activity was also observed for BAC 08H01 (Figure 2B), restricting the minimal genetic interval for *pri* function to a 52.6-kb DNA sequence (light yellow, Figure 2A).

These data, therefore, show that despite a compact transcribed region, the developmental functions of *pri* relies on the activity of large upstream and downstream genomic sequences.

### Multiple Enhancers Drive Tissue-Specific Expression of *pri* in Embryos

**FIGURE 3:**
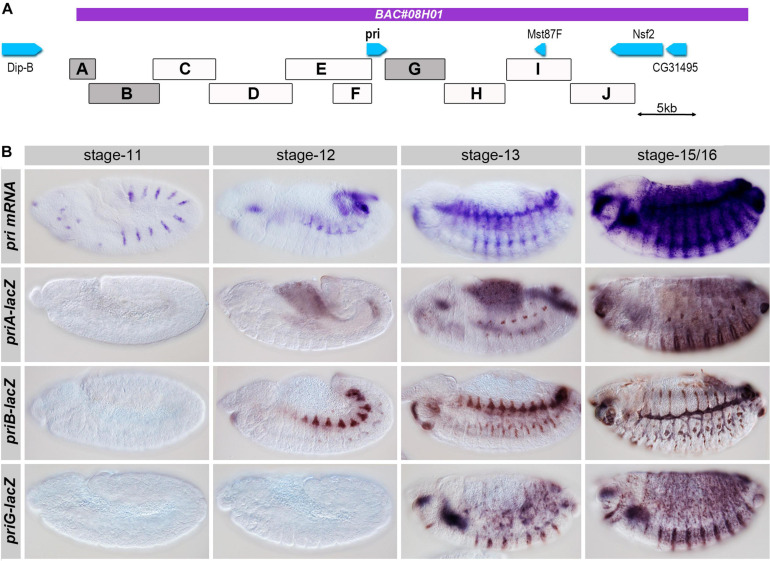
Functional identification of *pri* embryonic enhancers. **(A)** Boxes represent the genomic location of the regions tested using lacZ-reporter transgenic lines; the boxes highlighted in gray showed a significant activity during embryogenesis, and the purple line shows the region carried by BAC08H01 that restores significant adult viability. **(B)** Time course of *pri* mRNA expression from mid to late embryogenesis, as revealed by *in situ* hybridization (top). Expression driven by the three main embryonic enhancers (*priA*, *priB*, and *priG*) at corresponding stages of development was revealed by anti-ß-Gal immunostaining.

Having defined the functional genetic unit of *pri* for *Drosophila* development, we next attempted to delineate the *cis-*regulatory sequences controlling its expression in embryo. We used a series of transgenic LacZ reporter lines (Chanut-Delalande et al., 2014) to systematically examine the whole region. The *pri* locus was dissected in DNA fragments of approximately 5 kb long, which displayed overlapping regions to ensure coverage of the locus (Figure 3A). We performed LacZ staining on staged embryos to define the spatial and temporal pattern(s) of expression driven by each region. Three regions drove expression in subsets of embryonic cells, reproducing parts of the endogenous pattern of *pri* mRNA. *priG* was expressed in the epidermis from stage 13 mostly in ventral cells, and its activity extended to the dorsal region in stage 15/16 (Figure 3B). A second remote enhancer, *priA*, also exhibited epidermal expression in the dorsal and ventral regions. Following a faint onset of epidermal activity at stage 13, *priA* displayed a strong activity in the epidermis, which peaked at stage 15/16 (Figure 3B). Both *priA* and *priG* enhancers displayed stronger activity in presumptive trichome cells (as best seen in the ventral region), consistently with the endogenous pattern of *pri* mRNA (Galindo et al., 2007; Kondo et al., 2007). Finally, we observed that the *priB* enhancer drove strong expression in the embryonic tracheal system from stage 12–16 (Figure 3B). This activity was yet slightly delayed when compared with *pri* endogenous expression in the tracheal system, the onset of which being visible at stage 11. Other DNA regions, including those comprising the *pri* promoter, displayed either barely detectable activity or expression in tissues (posterior gut, groups of mesodermal cells, hemocytes, see Supplementary Figure 1) that might reveal additional aspects of *pri* expression, but which currently lack functional evidence.

All together, these results identify three main enhancers that control *pri* expression across embryonic development. Two separate *cis-*regulatory regions, *priA* and *priG*, drive *pri* expression in the epidermis and a third one, *priB*, in the tracheal system.

### Ecdysone Controls the Activity of Separate *pri* Embryonic Enhancers

Previous work has shown that the expression of *pri* in the embryonic epidermis requires ecdysone signaling (Chanut-Delalande et al., 2014). Since the embryonic expression of *pri* is driven by three major enhancers, *priA*, *B*, and *G*, we next investigated whether these enhancers could be regulated by ecdysone and EcR.

**FIGURE 4:**
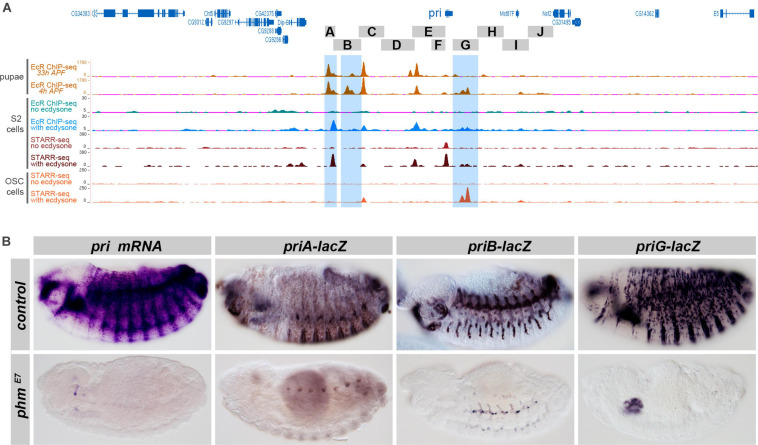
Ecdysone controls *pri* expression and the activity of embryonic enhancers. **(A)** Genome browser snapshot of the genomic region encompassing the *pri* locus; *pri* regions tested in transgenic lacZ-reporter lines are drawn as gray boxes. The different tracks represent signal intensity profiles of *in vivo* EcR ChIP-Seq [4 and 33 h after puparium formation (APF)] in orange (Chanut-Delalande et al., 2014), EcR ChIP-seq in S2 cells with or without ecdysone treatment (blue and green, respectively), STARR-seq profiling of active enhancers with or without ecdysone (brown and dark red) in S2 cells, as well as STARR-seq profiling with or without ecdysone (red) in ovarian somatic cells (OSC) (Shlyueva et al., 2014). **(B)** Endogenous expression of *pri* mRNA, and of *priA*, *priB*, and *priG* enhancers, in control conditions and *phm*^*E7*^ mutant embryos, which are unable to synthesize ecdysone (Warren et al., 2004).

ChIP-seq data showed that the *pri* locus is prominently bound by EcR in pupae (Chanut-Delalande et al., 2014), and each of the three embryonic enhancers *priA*, *priB*, and *priG* contain major EcR peaks (highlighted in blue in Figure 4A). In addition, profiling of EcR binding in cultured S2 cells (Shlyueva et al., 2014) also detected a strong EcR peak in the *priA* sequence. Although displaying a weaker intensity, EcR binding is also seen within *priB* and *priG* sequences. These EcR binding events are likely to be relevant, since the signal is only seen in the presence of ecdysone (Figure 4A). The same study used high-throughput STAR-seq profiling to functionally define enhancers whose activity was specifically activated upon ecdysone treatment in cultured cells (Shlyueva et al., 2014). Of note, the *priA* region contains the strongest ecdysone-responsive enhancer of the third chromosome in S2 cells. A weaker enhancer activity was also found in the *priG* region in S2 cells, while *priG* corresponded to a major ecdysone-responsive enhancer (Figure 4A) in OSC cells (Shlyueva et al., 2014).

During embryogenesis, the expression of *pri* mRNA in the tracheal system, epidermis, gut, and pharynx was abolished in *phantom* (*phm)* mutant embryos (Figure 4B), which are unable to synthesize ecdysone (Warren et al., 2004). We next tested whether the lack of ecdysone also influenced the activity of *pri* embryonic enhancers. In embryos deprived of ecdysone, the activity of *priG* was strongly impaired, preventing the detection of any epidermal expression (Figure 4B). As an additional test, we drove a dominant negative version of EcR (EcR^DN^) in a subset of epidermal cells using *ptc*-gal4, leading to a strong reduction of *priG* epidermal expression in EcR^DN^ cells, as also observed for endogenous *pri* mRNA (Supplementary Figure 2). The activity of *priA* was also dramatically decreased in the absence of ecdysone (Figure 4B). Finally, although not entirely abolished, expression of the *priB* enhancer was reduced in the tracheal system (Figure 4B). In contrast, other *pri* regions that contain major pupal peaks of EcR binding in pupae (*priBC*, *priDE*) only displayed weak embryonic expression without obvious reduction in *phm* mutant embryos (Supplementary Figure 3).

Taken together, these data indicate that ecdysone is a requisite for the activity of *priA* and *priG* epidermal enhancers in the embryo, and to a lesser extent of the tracheal enhancer *priB*.

### EcR Binds to *pri* Enhancers in a Tissue-Specific Manner

Although the 20E/EcR complex regulates the expression of many genes (Li and White, 2003; Beckstead et al., 2005; Gauhar et al., 2009; Shlyueva et al., 2014; Stoiber et al., 2016; Uyehara and McKay, 2019), experimental evidence of its direct role in the *in vivo* activity of enhancers remains limited. We aimed to get a deeper understanding of *pri* regulation in the epidermis by ecdysone, and the *priA* and *priG* enhancers appeared as good candidates for direct regulation by EcR.

**FIGURE 5:**
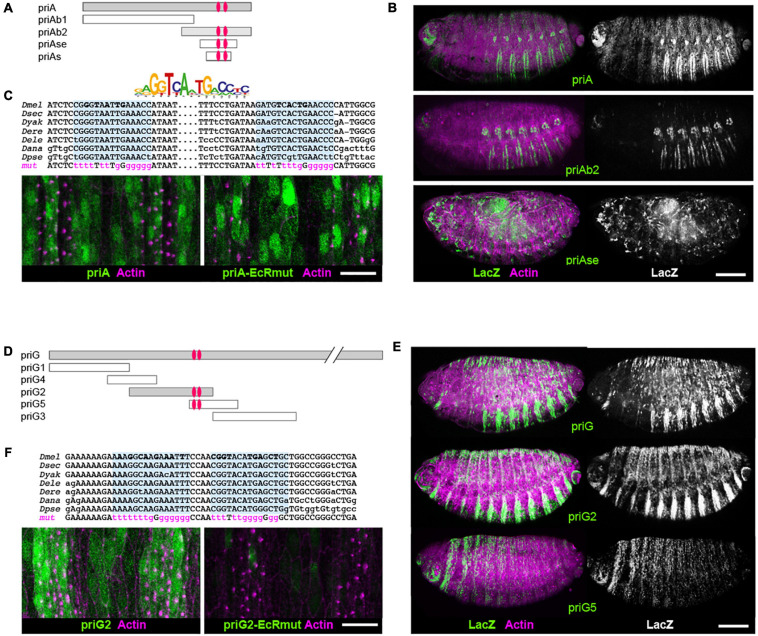
Ecdysone nuclear receptor (EcR) binding sites are required for the activity of *pri* epidermal enhancers. **(A)** Scheme of the *priA* enhancer and its additional subregions that have been tested in lacZ-transgenic reporter lines. Red ovals represent the EcR binding sites shown in panel **(C)**. **(B)** Expression of *priA*, *priAb2*, and *priAse*, as revealed by fluorescence staining (ß-gal is in green, actin in magenta); the right panels show black and white pictures of the ß-gal channel for better contrast. All pictures have been acquired using the same setups. Scale bar is 100 μm. **(C)** The logo represents position weight matrix of consensus EcR binding sites. Sequence alignment of EcR binding sites in *priA*, showing their evolutionary conservation across *Drosophila* species (*melanogaster*, *sechellia*, *yakuba*, *erecta*, *elegans*, *ananassae*, and *pseudoobscura*). Nucleotides mutated in *priA-EcRmut* are in magenta. Bottom pictures show confocal microscopy images of the ventral epidermis in stage 15 embryos. GFP (green) reveals the expression of *priA* and *priA-EcRmut* enhancers; nascent trichomes accumulate F-actin (magenta) microfilaments. Scale bar is 10 μm. **(D)** Drawing of the molecular dissection of the *priG* epidermal enhancer. **(E)** Expression of *priG-lacZ*, *priG2-lacZ*, and *priG5-lacZ* as revealed by fluorescence staining (ß-gal is in green, actin in magenta). Scale bar is 100 μm. **(F)** Sequence alignment of EcR binding sites of *priG2* (top) and confocal microscopy images (bottom) that show the effect of their mutation (*priG2-EcRmut*) on the enhancer activity (ventral epidermis close up). GFP is in green, F-actin in magenta. Scale bar is 10 μm.

We carried out systematic dissection of these two enhancers to delineate the respective minimal region sufficient to drive proper expression in embryonic epidermis. The *priA* element (Figures 5A–C) contains a sequence of 288 bp that drives ecdysone-dependent expression in S2 cells and whose activity was abolished following the mutation of two EcR binding sites (Shlyueva et al., 2014). These two sites are evolutionarily conserved across *Drosophila* species (Figure 5C) and a 3′ deletion of *priA* that lacks this region, *priAb1*, was devoid of activity in embryos (not shown). Reciprocally, the *priAb2* region displayed epidermal expression, although with a significantly decreased intensity when compared with the full *priA* enhancer (Figure 5B). However, the short enhancer delineated in S2 cells (*priAs*) was not sufficient to drive a proper *in vivo* expression in the embryonic epidermis. Instead, we observed ectopic expression in scattered cells that likely represents blood cells. Similar results were also observed with a construct bearing an extended version *priAse* (421 bp, Figures 5A,B). These data indicate that the *in vivo* activity of *priA* requires additional *cis-*regulatory elements present in the 5′ region of the *priA* sequence, which may include other EcR binding sites. Accordingly, mutation of both EcR binding sites in the backbone of the full *priA* enhancer did not significantly impact on its epidermal expression (Figure 5C).

The *priG* enhancer was also dissected in a series of overlapping DNA fragments of approximately 1 kb in length (*priG1-5*, Figure 5D). Transgenic reporters displayed no significant activity for *priG1* or *priG4*, *priG3* and *priG5* displaying only weak expression in embryos (Figure 5E). In contrast, *priG2* exhibited strong epidermal activity comparable with the entire *priG* enhancer. Using the JASPAR database of transcription factor binding sites (Mathelier et al., 2014), we found two juxtaposed putative EcR sites in *priG2*, which are evolutionarily conserved (Figure 5F). We inactivated these EcR binding sites in the *priG2* construct by site-directed mutagenesis, and the disruption of both sites abolished the activity of the mutated enhancer, *priG2-EcRmut*, when assayed in embryos (Figure 5F).

Hence, we identified a 1-kb minimal regulatory region, *priG2*, sufficient to drive expression in epidermal cells. Its epidermal activity is dependent on ecdysone signaling, likely through a direct action of EcR since the mutation of EcR binding sites is sufficient to inactivate this enhancer.

### Distinct Enhancers Drive *pri* Expression in the Leg Primordium

**FIGURE 6:**
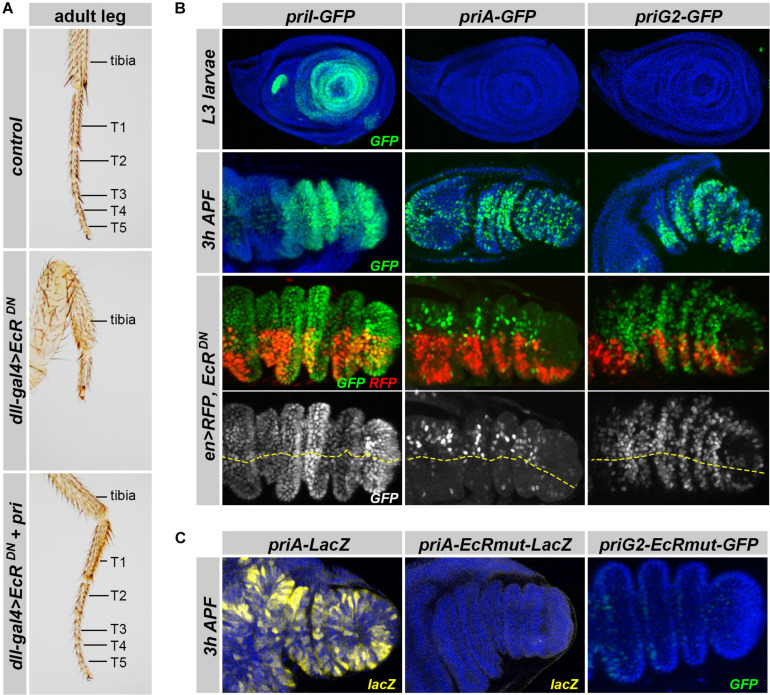
Expression of *pri* enhancers active in leg primordia at the larval/prepupal transition. **(A)** Pictures of adult forelegs in control individuals or animals expressing EcR^DN^ under the control of *dll-gal4*, with or without simultaneous expression of *pri* mRNA. T1–5 represents tarsal segments. **(B)** Activity of *priI*, *priA*, and *priG2* enhancers (green) in control leg disks (upper panels) at mid L3 and pupal (3–5 h APF) stages (two top panels). The two bottom panels show the effect of expressing EcR^DN^ under the control of *en-Gal4* [that also drives RFP (red)] on the activity of *priI*, *priA*, and *priG2* enhancers in pupal leg disks. DAPI is in blue. The yellow line highlights the boundary between RFP-positive (posterior compartment) and RFP-negative (anterior) cells. **(C)** Effect of inactivating EcR binding sites on the activity of *priA* (ß-Gal immunostaining, yellow) and of *priG2* (GFP, green) in the pupal leg primordium. Nuclei are visualized by DAPI (blue).

During post-embryonic development, *pri* is essential for adult leg morphogenesis, and the loss of *pri* leads to strong defects in tarsal segments, which fuse and do not develop (Galindo et al., 2007; Pueyo and Couso, 2008, 2011; Pi et al., 2011). *Pri* expression in the leg disk is controlled by ecdysone (Chanut-Delalande et al., 2014), and we assayed whether EcR function was required for tarsal development. As expected, driving EcR^DN^ in the distal region of legs using *dll-gal4* provoked strong phenotypes, in which tarsal segments were mostly absent with only a small region likely representing atrophic and fused remnants of those (Figure 6A). Importantly, we found that *pri* was sufficient to suppress the defects resulting from EcR^DN^ expression, therefore, suggesting that *pri* is a major target of ecdysone signaling for adult appendage development.

Using *in vivo* reporter assays, we next searched for genomic regions driving *pri* expression during the formation of adult structures, which occurs from larval to pupal stages. To monitor the activity of *pri* enhancers during post-embryonic development, we generated a series of transgenic lines that drive a nuclear-GFP reporter. We did not observe expression for *priC*, *DE*, *F*, *H*, and *J* enhancers in the leg primordium, neither in the third instar imaginal disks nor after puparium formation (Supplementary Figure 4 and data not shown). In contrast, five separate *pri* enhancers (*priA*, *priB*, *priBC*, *priG2*, and *priI*) drove significant expression in leg disks, with a marked temporal regulation of their activity. Notably, *priI* drove expression in the larval leg disk from the early-mid third instar stage (L3), which was then downregulated in late L3, and came up with a high expression from 1 to 6 h after puparium formation (APF) (Figure 6B), i.e., a pattern reminiscent of the endogenous expression of *pri* mRNA (Galindo et al., 2007; Pueyo and Couso, 2008, 2011; Chanut-Delalande et al., 2014). *priB* displayed a very limited expression in larval leg disks, which was substantiated at pupal stages (Supplementary Figure 4). *priA*, *priBC*, and *priG2* enhancers, which were not active in larval leg disks, drove salt-and-pepper patterns of expression in pupal legs (Figure 6B and Supplementary Figure 4).

To decipher how *pri* expression was regulated by ecdysone signaling, we tested whether the loss of EcR function affected the expression of *pri* enhancers in leg disks. We used targeted expression of EcR^DN^, or *EcR-RNAi*, driven in the posterior compartment by *en-Gal4*, the anterior region providing internal control (Figure 6B and Supplementary Figure 4). Although some *pri* enhancers were not (*priI*) or only slightly (*priG2*) affected by EcR inactivation (Figure 6B and Supplementary Figure 4), the activity of *priA* was clearly reduced in the posterior cells of pupal leg disks, when compared with wild-type anterior cells. Furthermore, point mutations that disrupt the two EcR binding sites (required in S2 cells but not in embryos, see Figure 5) were sufficient to abrogate *priA* activity in pupal legs (Figure 6C). Of note, we found that the *priG2* enhancer in which the binding sites for EcR have been killed also displayed reduced activity in the pupal leg disks, suggesting that our EcR^DN^ conditions represented only a partial inactivation of ecdysone signaling.

**FIGURE 7:**
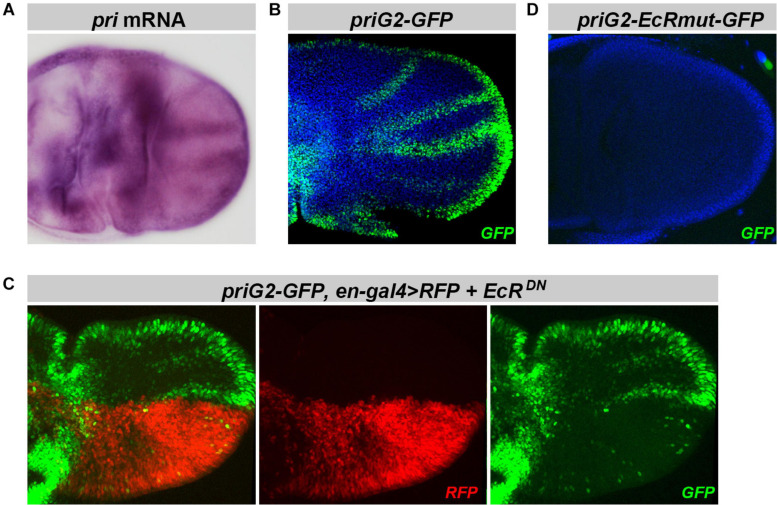
Expression of *priG2* in the pupal wing primordia is controlled by EcR. **(A)** Picture of *in situ* hybridization showing that *pri* is expressed in the pupal wing primordia (3–5 h APF), with a stronger signal in the presumptive regions of adult veins [see also Pi et al. (2011)]. **(B)** *pri-G2* displays a pattern reproducing *pri* mRNA distribution in the wing disk. **(C)** Expression of EcR^DN^ under the control of *en-gal4* (monitored by the expression of RFP, in red) decreases *priG2* activity in corresponding posterior cells. **(D)** Point mutations that inactivate both putative EcR binding sites within the *priG2* sequence abrogate *priG2* activity in pupal wing disks. GFP is shown in green, RFP in red, and DAPI is in blue.

*Pri* function is also required for the proper development of adult wings (Pi et al., 2011). Soon after puparium formation, *pri* is highly expressed in wing disks (Pi et al., 2011) and accumulates in L2–L5 provein regions (Figure 7A). Among *pri* enhancers, we found that *priG2* drove a pattern mimicking endogenous expression of *pri* mRNA (Figure 7B and Supplementary Figure 5). In the pupal wing, the activity of *priG2* was clearly decreased by EcR^DN^ expression (Figure 7C). Furthermore, inactivation of EcR binding sites abrogated activity of the *priG2* enhancer in pupal wing disks (Figure 7D). We concluded that *priG2* is a major enhancer that drives *pri* expression in the wing, likely under direct control of EcR.

Taken together, these data show that *pri* expression is driven by multiple enhancers during the morphogenesis of adult tissues, with one, *pri*A, directly responding to EcR in developing legs, while one other, *priG2*, requires EcR activity in the wing primordium.

## Discussion

**FIGURE 8:**
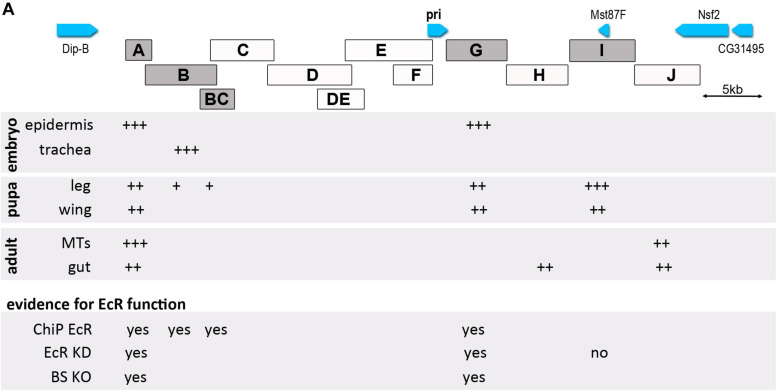
Schematic representation of *pri* enhancer activity along *Drosophila* development. The detected activity of pri enhancers is shown by a + sign, their number depending on the intensity of the activity. Data for embryo and pupa are from this paper and from Bohère et al. (2018) and Al Hayek et al. (2021) for adult tissues [adult stem cells of Malpighian tubules (MTs) and of the gut]. For positive enhancers, we visualized EcR binding (ChiP EcR), effect of EcR function depletion on enhancer activity (EcR KD) and activity of enhancers mutated on specific EcR binding sites (BS KO).

Our data show that the *pri* gene encoding smORF peptides is a major core component of the response to ecdysone. We found that *pri* expression involves a wide array of enhancers, some of them being likely directly controlled by the ecdysone nuclear receptor EcR (Figure 8). These results further suggest that the *cis-*regulatory architecture of the *pri* gene contributes to shape tissue- and stage-specific patterns of ecdysone response during development.

### The Pri Gene Displays Hallmarks of a Master Developmental Gene

The expression of eukaryotic genes is primarily determined by the activity of proximal promoter elements, whose activity is further regulated by enhancers that can act at a distance. Genes can schematically be separated in two classes, those initiating transcription over a broad region (dispersed transcription) and generally expressed in most cell types, and those initiating transcription at a focused position, which often involves a TATA box element and remote regulatory enhancers, such as important developmental genes (Vo Ngoc et al., 2017; Furlong and Levine, 2018).

Contrasting with the compact size of its transcribed region, *pri* functions throughout development involve an unexpectedly large genomic locus, as defined by rescuing assays. The smallest DNA fragment that allows full rescue of *pri* activity spans over 50 kb, indicating that *pri* expression relies on a large array of *cis-*regulatory regions scattered over this region, which underlie the highly dynamic expression of *pri* across tissues and developmental stages (Savard et al., 2006; Galindo et al., 2007; Kondo et al., 2007). Consistent with this conclusion, we identify remote enhancers located as far as 20 kb upstream and 10 kb downstream of the *pri* transcriptional unit. The attribution of *cis-*regulatory elements located within large intergenic regions to their respective target genes yet remains difficult. For example, the *priI* enhancer overlaps with *Mst87F*, a male-specific transcript expressed in the germline from late pupal stages onward (Kuhn et al., 1991). The remote *priI* enhancer, nevertheless, reproduces the expression of *pri* in the developing larval/pupal leg disk. Also, the strong EcR binding peak contained in the *priA* enhancer was previously attributed to *Dip-B* (Shlyueva et al., 2014), which is located closer than *pri*. Several pieces of evidence are, however, consistent with its role in the regulation of *pri* expression. First, *priA* drives a stage-specific embryonic expression in epidermal cells like *pri* mRNA, while *Dip-B* is mostly expressed in the hindgut and Malpighian tubules^3^. Second, *priAb2* that exhibits epidermal expression is contained in the smaller *pri* rescuing BAC. Third, levels of *pri* mRNA induction-triggered ecdysone treatment is several times higher than for *Dip-B*, as seen in Kc cells (Skalska et al., 2015), well in line with the strength of this ecdysone-responsive element (Shlyueva et al., 2014). It remains possible that this enhancer contributes, at least in part, to the control of *Dip-B* expression. The *priA* region also represents an interesting case to compare the function of an enhancer between cultured cells and *in vivo* tissues. While *ex vivo* assays have demonstrated the strong activity of a short region of *priA* directly bound by EcR (Shlyueva et al., 2014), neither this sequence nor an extended version of it is sufficient to faithfully drive *pri* (or *Dip-B*) expression in the embryo. Instead, it leads to ectopic expression in blood cells. It is interesting to note that the activity of this short element in S2 cells also requires binding sites for GATA factors (Shlyueva et al., 2014), and the GATA factor Serpent is well known to play a key in the blood cell lineage (Rehorn et al., 1996; Muratoglu et al., 2007).

Distinct *pri* enhancers can drive similar and/or overlapping patterns, for example, *priA* and *priG* in the embryonic epidermis, or *priI*, *priA*, *prig*, and *priB* in the leg primordium. The expression of major developmental genes often involves apparently redundant enhancers, sometimes called shadow enhancers, which ensure a robust spatiotemporal gene expression, in particular, when development proceeds under non-optimal environmental conditions or in compromised genetic backgrounds (Frankel et al., 2010; Perry et al., 2010; Osterwalder et al., 2018). Genome-wide studies revealed that many developmental genes have shadow enhancers driving a similar activity (Menoret et al., 2013; Cannavo et al., 2016). Therefore, multiple enhancers active in the same tissue could collectively provide robustness against genetic and/or environmental variations to ensure the proper expression of *pri*, throughout embryonic and post-embryonic development.

Individual *pri* enhancers can also be active in different tissues and/or developmental stages; this feature is called pleiotropy (Preger-Ben Noon et al., 2018). *PriA* represents a striking case of a pleiotropic enhancer that is expressed in both the embryonic epidermis and the leg primordium, as well as in renal (Bohère et al., 2018) and intestinal (Al Hayek et al., 2021) adult stem cells. Of note, other genes of the ecdysone cascade possess pleiotropic enhancers, as seen with *EcR*, *Eip75B*, and *ftz-f1* that are driven by enhancers active in both somatic and germline tissues during oogenesis (McDonald et al., 2019). Pleiotropic enhancers may serve as a platform to drive gene expression in several spatiotemporal contexts (Sabaris et al., 2019), and future work will be required to better understand their behavior and functions during development (Preger-Ben Noon et al., 2018).

### Pri Is a Core Element of the Response to Ecdysone

Animal development requires a precise temporal coordination, across the whole organism, of genetic programs underlying the formation of the different tissues and organs, as well exemplified by major morphological changes such as the transition of immature juveniles into reproductive adults (Rougvie, 2001). The steroid hormone ecdysone plays a key role in the temporal control of major developmental transitions, acting as a systemic signal timely released in response to various inputs from both the internal milieu and the environment (Ou and King-Jones, 2013; Yamanaka et al., 2013). While we now have a wealth of information on the regulation of ecdysone production (Andersen et al., 2013; Ou and King-Jones, 2013; Yamanaka et al., 2013; Niwa and Niwa, 2014), how ecdysone signaling is integrated within terminal differentiation programs remains to be fully elucidated.

Previous work on Pri peptides has provided a molecular connection between the intimate mechanisms of epidermal differentiation and ecdysone signaling (Chanut-Delalande et al., 2014). Several arguments support that *pri* is directly activated by hormone-bound EcR. First, the expression of *pri* is abrogated in mutant embryos lacking ecdysone. Of note, the inactivation of any enzyme of the ecdysone biosynthesis pathway causes a similar phenotype, referred to as Halloween (Warren et al., 2002, 2004; Petryk et al., 2003; Gilbert, 2004; Niwa et al., 2004, 2010; Namiki et al., 2005; Ono et al., 2006; Chanut-Delalande et al., 2014; Enya et al., 2014), which is characterized by a poorly differentiated cuticle and the complete lack of trichomes. Second, there are prominent peaks of EcR binding to *pri* genomic regions *in vivo* (Chanut-Delalande et al., 2014), as well as in different cells lines following ecdysone treatment that induces a burst of *pri* transcription (Shlyueva et al., 2014; Skalska et al., 2015). RNA-seq data from cell lines also provide information on the kinetics of *pri* induction, which starts after that of *Eip75B* and *Hr4*, therefore, suggesting that the activation of *pri* expression requires both 20E/EcR binding and the production of early TFs, which is representative of the so-called early–late response genes (Ou and King-Jones, 2013). When assayed *in vivo*, several regions of the *pri* locus act as tissue-specific enhancers responsive to ecdysone during development. The activity of *priA*, *priB*, and *priG* enhancers is compromised in embryos lacking ecdysone, and targeted expression of EcR^DN^ represses the expression of *priG* and *priA* enhancers in corresponding cells, in embryos and larval/pupal tissues. Finally, point mutations of EcR binding sites are sufficient to kill the activity of *priG2* and *priA*, in the embryo and in pupal wings or legs, as expected if the transcription of *pri* involves a direct control by the 20E/EcR complex.

Our results further suggest that *pri* is a key and broad mediator of the response to ecdysone. Indeed, the re-expression of *pri* is sufficient to compensate for deficient ecdysone signaling across various tissues. In embryos, *pri* expression in epidermal cells restore trichomes in the complete absence of ecdysone, showing that *pri* is a pivotal target of ecdysone action in the epidermis (Chanut-Delalande et al., 2014). The same is also true in tracheal cells, as shown recently (Taira et al., 2021). In a similar way, the expression of Pri peptides can counteract the defects resulting from targeted expression of EcR^DN^ in pupae, restoring both proper development and adult viability (Chanut-Delalande et al., 2014). Recent studies have revealed that ecdysone signaling is essential to adapt the behavior of intestinal stem cells in adult females, as part of a post-mating response (Ahmed et al., 2020; Zipper et al., 2020). We found that Pri peptides are playing an important role in this ovary-to-gut interorgan communication, being able to substitute for ecdysone signaling in intestinal stem cells (Al Hayek et al., 2021). Hence, Pri peptides appear as key mediators of ecdysone, in a broad variety of tissues throughout development, as well as in adult stem cells. We propose that *pri* may belong to a small number of genes, representing a core module for the response to ecdysone. Supporting this model, *pri* expression is strongly induced by ecdysone in almost all cell lines that have been profiled by the work of Stoiber et al. (2016), presenting a behavior similar to the canonical *Broad* and *Eip74EF* factors. Future work will be needed to thoroughly test the full extent of Pri peptides in mediating response to ecdysone during development and adulthood.

### Pri *Cis-*Regulatory Architecture Contributes to Shape Stage and Tissue-Specific Patterns of the Response to Steroids

One most intriguing question about ecdysone signaling is how this systemic hormone can trigger various patterns of tissue-specific responses, including different timing across target tissues. Several mechanisms are likely at work in implementing specific responses to ecdysone. They include regulation of ecdysone uptake in peripheral tissues (Okamoto et al., 2018; Okamoto and Yamanaka, 2020), the existence of distinct EcR isoforms with tissue-specific functions (Cherbas et al., 2003; Braun et al., 2009; Gautam et al., 2015), the variation of partners associating with EcR [e.g., ultraspiracle that generally dimerizes with EcR (Hodin and Riddiford, 1998) is not required for activating glue genes in salivary glands (Costantino et al., 2008)], as well as chromatin factors that regulate accessibility of EcR binding sites across cell types (Gutierrez-Perez et al., 2019). Since *pri* appears required for the response to ecdysone in various cell contexts, the dynamic pattern of *pri* expression might also contribute to determine which and when tissues become competent to respond to the systemic hormonal signal.

To explain how *pri* is activated by ecdysone in so many different contexts, a simple model would be that EcR might act on *pri* promoter and/or proximal elements, distant enhancers providing tissue-specific regulatory cues. Instead, our results indicate that independent remote *pri* enhancers likely require the direct binding of EcR for their activity, since their activity is abolished upon the mutation of EcR binding sites. Therefore, the *cis-*regulatory landscape of *pri* that involves multiple enhancers appears well suited to sculpt various patterns of ecdysone response throughout development. If, as supported by the current data, *pri* is a major mediator of ecdysone, one would expect that changes within the *pri* locus may provide efficient means to adapt the organism to environmental conditions, at different time-scales throughout evolution. Although this hypothesis remains to be tested, it is interesting to note that *pri* has been identified as a candidate gene to be involved in the adaptation to darkness (Izutsu et al., 2015).

## Conclusion

In this work, we depicted the complex regulation of the *pri* gene by ecdysone to sustain strong levels of expression that dynamically varies among tissues and along development. Such a complexity in transcription regulatory mechanisms is well established for developmental genes encoding regular proteins, but it was yet unknown for genes only encoding smORF peptides. Our data suggest that smORF peptide genes can behave as *bona fide* key developmental regulators, opening novel research paths for the functional exploration of this recently emerging family of genes.

## Data Availability Statement

The original contributions presented in the study are included in the article/Supplementary Material, further inquiries can be directed to the corresponding author/s.

## Author Contributions

HC-D, YK, and FP contributed to the conception and design of the study and wrote the first draft of the manuscript. SP helped in the student supervision. AM-F performed the computational analysis. AD, JZ, MG, DM, PV, SM-P, and HC-D contributed to the experimental work and interpretation. All authors read and approved the submitted version.

## Conflict of Interest

The authors declare that the research was conducted in the absence of any commercial or financial relationships that could be construed as a potential conflict of interest.

## Publisher’s Note

All claims expressed in this article are solely those of the authors and do not necessarily represent those of their affiliated organizations, or those of the publisher, the editors and the reviewers. Any product that may be evaluated in this article, or claim that may be made by its manufacturer, is not guaranteed or endorsed by the publisher.

## Footnotes

1
http://flystocks.bio.indiana.edu/

2
http://flypush.imgen.bcm.tmc.edu/pacmanfly/

3
http://insitu.fruitfly.org/cgi-bin/ex/insitu.pl
