# Supplementary material for: Pri smORF Peptides Are Wide Mediators of Ecdysone Signaling, Contributing to Shape Spatiotemporal Responses
Source: Front Genet. 2021 Aug 30;12:714152. doi: 10.3389/fgene.2021.714152 (PMC8435736; doi:10.3389/fgene.2021.714152)
Supplement: Supplementary file 1 [file Data_Sheet_1.docx]

Supplementary Material

# Supplementary Figures


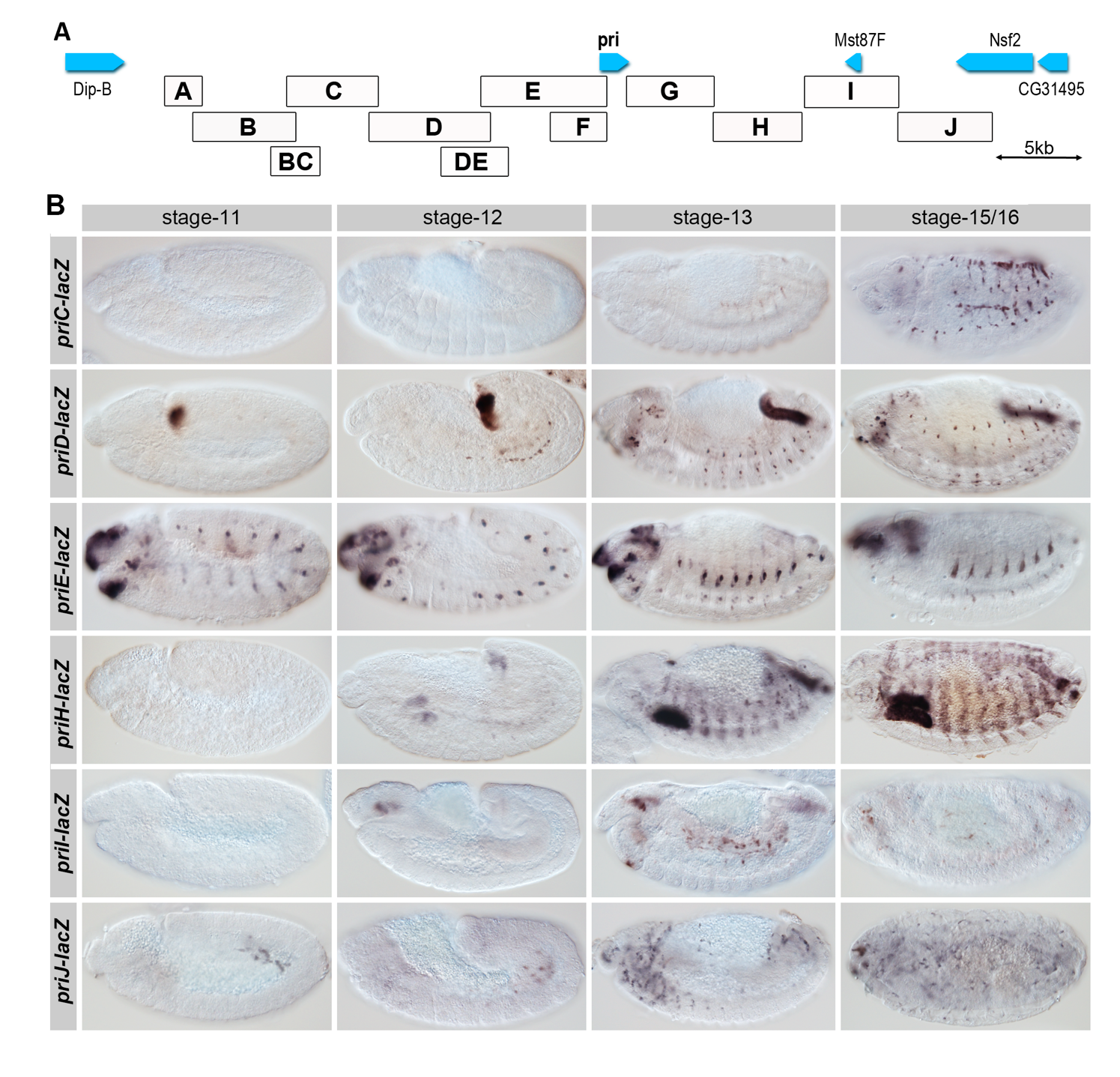


## Supplementary Figure 1. Characterization of pri enhancers with minor activity during embryogenesis. (A) Schematic representation of the *pri* locus. Genomic regions tested using *LacZ-*reporter lines are represented as white boxes. (B) Activity of *pri* genomic regions C, D, E, H, I and J from stage-11 to -16, showing weak expression and/or expression in other tissues than the epidermis and trachea.


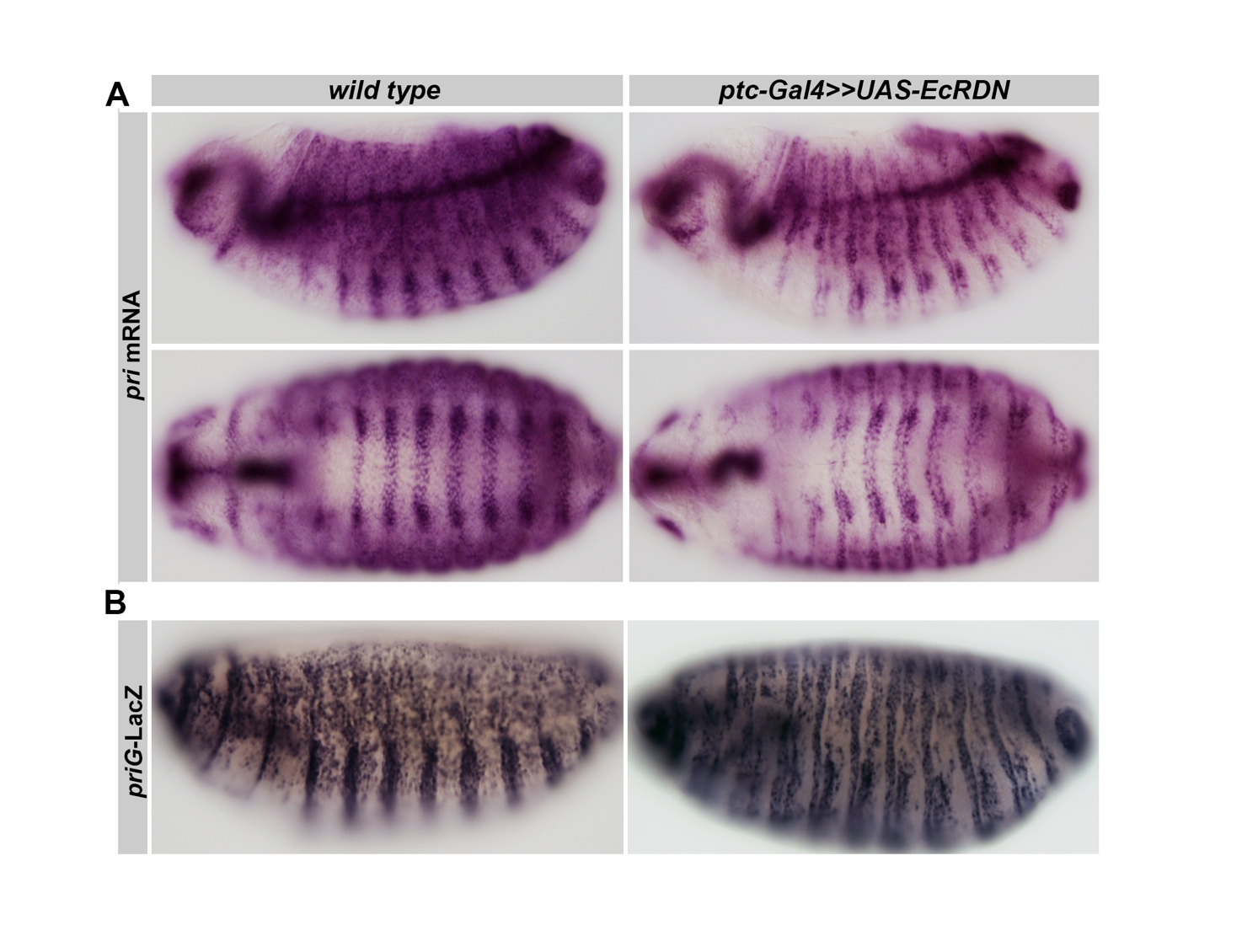


**Supplementary Figure 2.** Lack of ecdysone impairs *pri* expression and *pri*G activity. (**A)** *In situ* hybridization showing *pri* mRNA expression in wild type embryos (left) and embryos expressing EcR^DN^ driven by *ptc-Gal4* (right). pri expression is abolished in cell stripes expressing Ptc. Top panels show lateral views, bottom panels show ventral views. (**B)** LacZ immunostaining showing that *priG* activity is impaired in epidermal cells expressing EcR^DN^ (right) compared to control conditions (left).


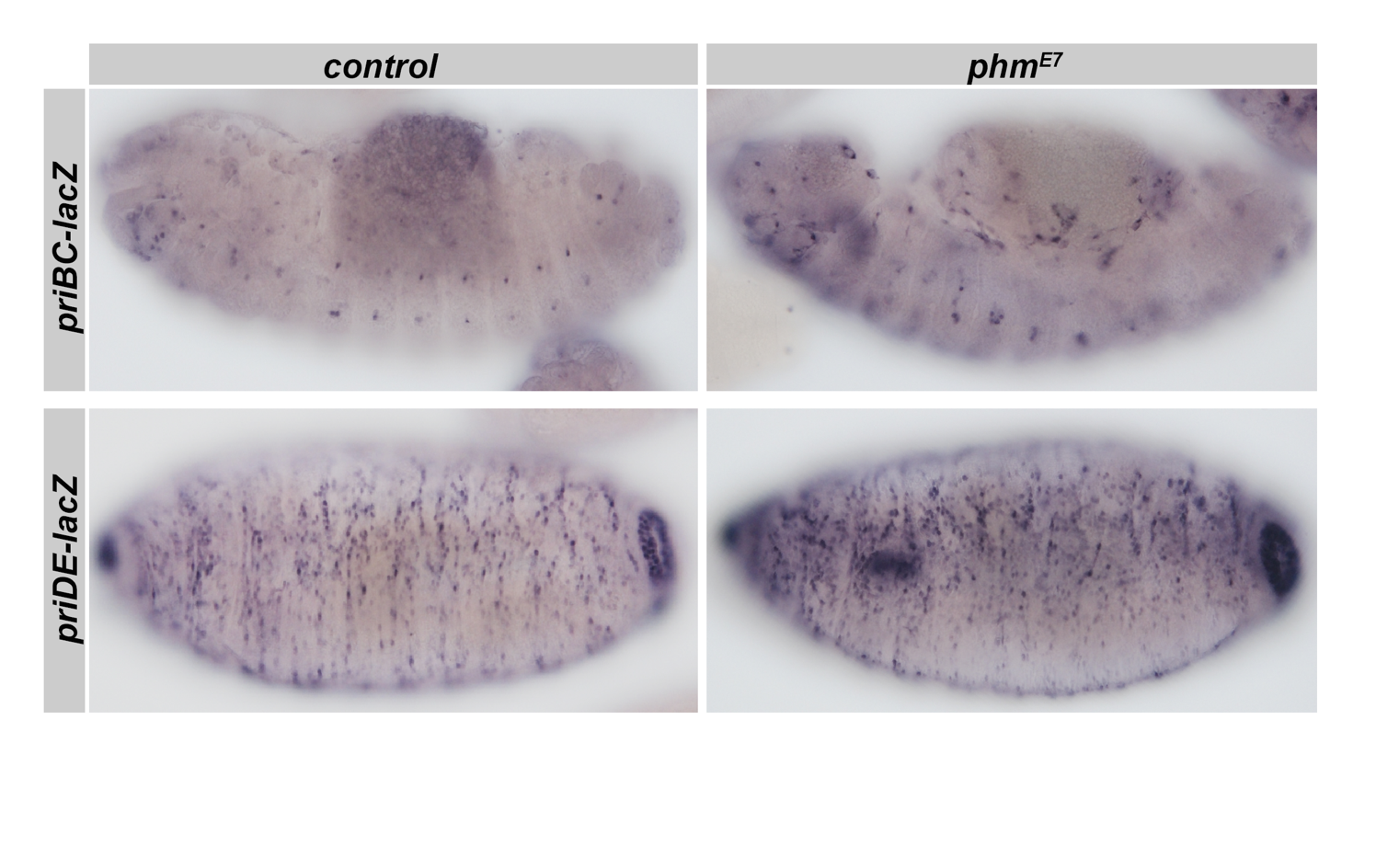


**Supplementary Figure 3.** Expression driven by *priBC* and *priDE* regions during embryogenesis. LacZ immunostaining show no obvious changes in *priBC* and *priDE* activity between *phm^E7^* mutant embryos deprived of ecdysone (right) and control sibling embryos (left). Pictures show stage-14 embryos for *priBC* and stage-16 embryos for *priDE*.


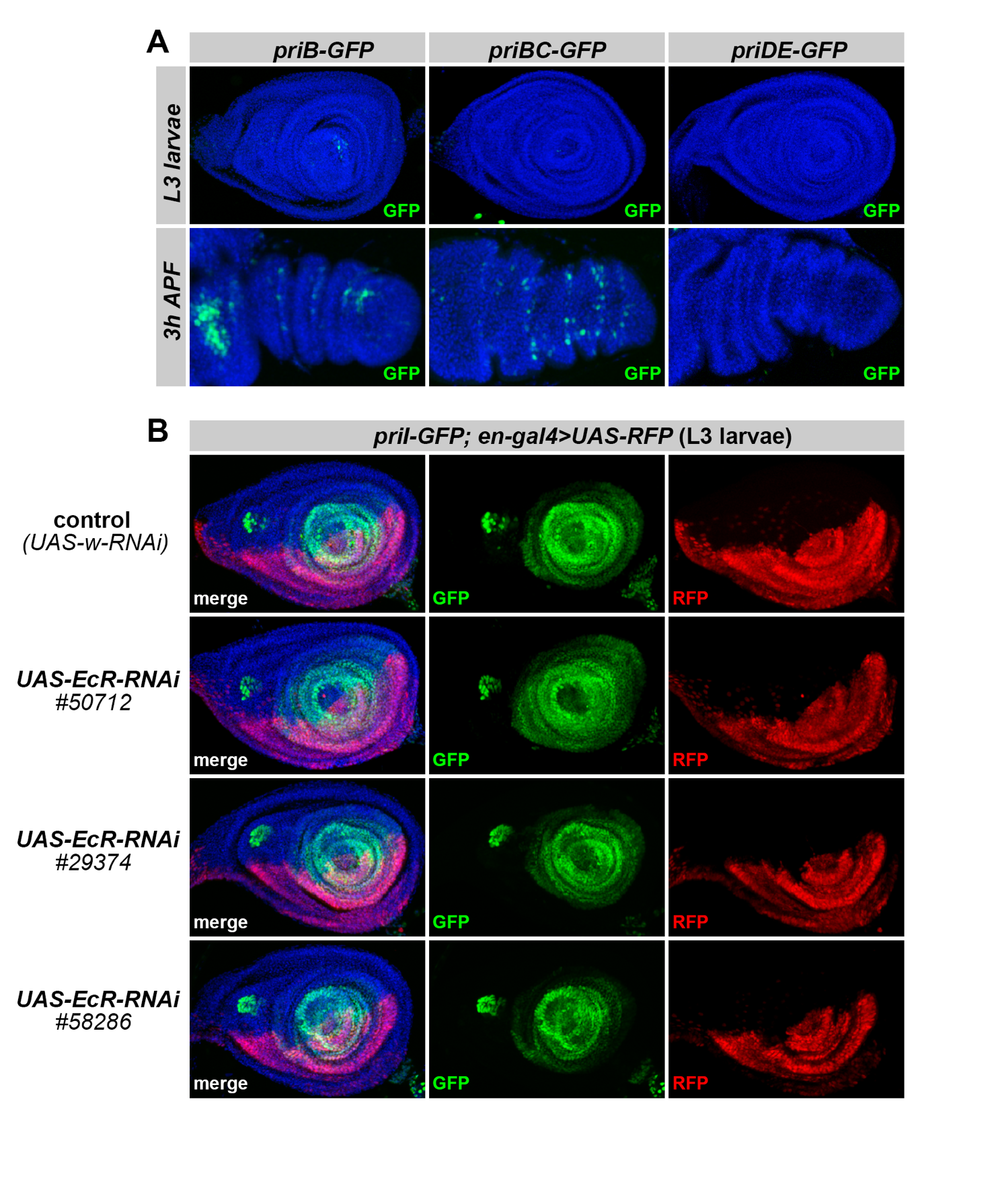


**Supplementary Figure 4.** (**A**) Expression of *priB*, *priBC* and *priDE* in the larval and pupal leg primordia. (**B**) Expression of *priI-GFP* in larval leg discs (mid-L3) in control conditions, or following concomitant expression of three different EcR-RNAi driven by *en-gal4* (monitored by RFP expression, red). Control individuals express an unrelated RNAi (directed against *w*). Blue is DAPI, and green is GFP.


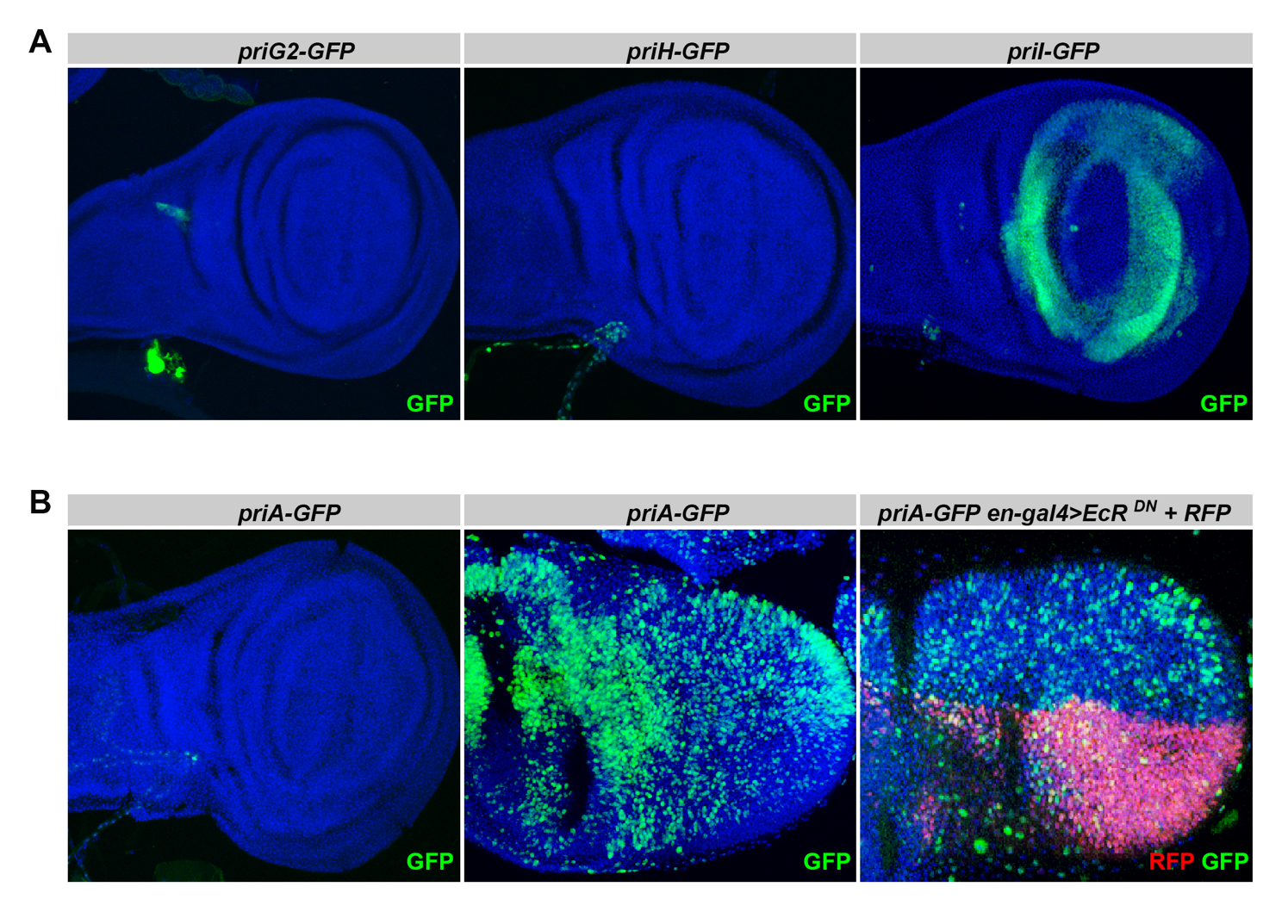


**Supplementary Figure 5.** Expression driven by *pri* enhancers in the wing imaginal disc. (**A**) expression of *priG2, priH* and *priI* in the wing larval imaginal discs in mid third instar larvae. (**B**) Expression of *priA-GFP* in larval (left panel) and pupal (right panels) wing discs in control conditions, or following concomitant expression of EcR^DN^ driven by *en-gal4* (monitored by RFP expression, red). The activity of *priA* is unchanged in cells expressing EcR-DN. In all pictures, blue is DAPI, green is GFP.

# Supplementary tables

| **constructs** | **start (3R:)** | **end (3R:)** | **size (bp)** |
| --- | --- | --- | --- |
| *priA* | 13 789 349 | 13 791 404 | 2 055 |
| *priA-EcR1mut* | 13 789 349 | 13 791 404 | 2 055 |
| *priA-EcR2mut* | 13 789 349 | 13 791 404 | 2 055 |
| *priA-EcRmut* | 13 789 349 | 13 791 404 | 2 055 |
| *priAb1* | 13 789 349 | 13 790 704 | 1 355 |
| *priAb2* | 13 790 601 | 13 791 404 | 803 |
| *priAse* | 13 790 753 | 13 791 174 | 421 |
| *priAs* | 13 790 835 | 13 791 161 | 326 |
| *priB* | 13 790 903 | 13 796 523 | 5 620 |
| *priC* | 13 796 024 | 13 801 009 | 4 985 |
| *priBC* | 13 795 378 | 13 798 178 | 2 800 |
| *priD* | 13 800 509 | 13 803 761 | 3 252 |
| *priE* | 13 806 614 | 13 812 824 | 6 210 |
| *priDE* | 13 805 078 | 13 808 878 | 3 800 |
| *priF* | 13 810 400 | 13 813 525 | 3 125 |
| *priG* | 13 814 566 | 13 819 339 | 4 773 |
| *priG1* | 13 814 566 | 13 815 557 | 991 |
| *priG2* | 13 815 540 | 13 816 559 | 1 019 |
| *priG2-EcRmut* | 13 815 540 | 13 816 559 | 1 019 |
| *priG3* | 13 816 536 | 13 817 556 | 1 020 |
| *priG4* | 13 815 297 | 13 815 880 | 583 |
| *priG5* | 13 816 279 | 13 816 862 | 583 |
| *priH* | 13 819 320 | 13 824 323 | 5 003 |
| *priI* | 13 824 304 | 13 829 359 | 5 055 |
| *priJ* | 13 829 348 | 13 834 473 | 5 125 |
| *bac1_CH321-08H01* | 13 790 547 | 13 843 138 | 52 591 |
| *bac2_CH322-176K10* | 13 796 285 | 13 816 692 | 20 407 |
| *bac3_CH321-51O01* | 13 782 785 | 13 881 493 | 98 708 |
| *bac4_CH322-150C08* | 13 807 968 | 13 829 890 | 21 922 |

**Supplementary Table 1.** Genomic position of transgenic constructs used in this study.

| ***construct*** | **name** | **sequence** |
| --- | --- | --- |
| *priA* | fwA3’hind3 | 5'-CGGATCCAAGCTTAGCAAATCAGTAATG-3' |
|  | rvA3' | 5'-GCGGAAATGCATTTCTATGGACTGACTTAC-3' |
| *priAb1* | fwA3’hind3 | 5'-CGGATCCAAGCTTAGCAAATCAGTAATG-3' |
|  | rvA3'block1 | 5'-ACACAAGTTCTAGACCACAAGTCATGCCAGAGCC-3' |
| *priAb2* | fwA3'block2 | 5'-TACAATCtAAGCTTGCGACAGGGAGGGCAGCATC-3' |
|  | rvA3'block2 | 5'-ATCCAATAGCGGCCGCAGTTATTAGTAAATGTATGC-3' |
| *priAse* | fwA3'S | 5'-TTCCGCAAAGCTTACACCACTAAAAAGTCCAAAAAGAAGG-3' |
|  | rvA3'S | 5'-GGTTTCAAGAATTCAGCATGAATATTCGG-3' |
| *priAs* | fwA3'ms | 5'-TTGTTTACAAGCTTGTTGTGGGGCGTTCGTTAGC-3' |
|  | rvA3'ms | 5'-TTAATTATGCGGCCGCGGAGACAATGCAAATGATTG-3' |
| *priA-EcR1mt* | gblockA3EcR1fw | 5'-GTAAGTCAGTCCATAGAAATGCATTTCCGC-3' |
|  | gblockA3EcR1rv | 5'-CAGAGTAACAGATAAGGCCATATCATGAC-3' |
| *priA-EcR2mt* | gblockA3EcR2fw | 5'-AGTCCTCTTCAGATCTGCTCAGTTGTTGC-3' |
|  | gblockA3EcR2rv | 5'-GGGGTTTCAAGAATTCAGCATGAATATTCG-3' |
| *priG* | priGfw | 5'-GACTCGAGGCGGCCGCTCGACCGACATCCAGAGAAC-3' |
|  | priGrv | 5'-GACTCGAGGCGGCCGCGTGTGTAGAGATGCTGATGC-3' |
| *priG1* | priG1fw | 5'-GACTCGAGGCGGCCGCTCGACCGACATCCAGAGAAC-3' |
|  | priG1rv | 5'-GATAAATTGCGGCCGCGTTCCTGGGAAGATCTGATTGCAGGC-3' |
| *priG2* | priG2fw | 5'-GATAAATTGCGGCCGCTCAGATCTTCCCAGGAACCCACACAC-3' |
|  | priG2rv | 5'-GATAAATTGCGGCCGCTTCATATACAATATAAACATTGATTCAGGCCC-3' |
| *priG3* | priG3fw | 5'-CGGACGCTCTAGAATCAATGTTTATATTGTATATGAATCGCCAGG-3' |
|  | priG3rv | 5'-GATAAATTGCGGCCGCTCATTGACCCTTTCTGCCTCGAGTTG-3' |
| *priG4* | priG4fw | 5'-GATAAATTGCGGCCGCAGAGAGACTATTAATAGACACGCGCGCAC-3' |
|  | priG4rv | 5'-GATAAATTGCGGCCGCACCAATTGAATCCCATAAGATATAAAAGGC-3' |
| *priG5* | priG5fw | 5'-GTAATTTCTAGAGTTCCAGCTCCAGCTCCCAACG-3' |
|  | priG5rv | 5'-GATAAATTGCGGCCGCGTGTAAATTGAACGCGGAGC-3' |
| *priG2EcRmut* | fwG2HindIII | 5'-CTAGCGGATCCAAGCTTGCATGCTG-3' |
|  | rvG2EcR1_EcR2mt | 5'-TTGGCCCCCCCCAAAAAAATCTTTTTTCATTTCTCAGAGCG-3' |
|  | fwG2EcR1_EcR2mt | 5'-TTTTTTTGGGGGGGGCCAATTTTTTGGGGGGGGCTGGCCGGGCCTGAATCAATG-3' |
|  | rvG2BSU36I | 5'-CAGTATCGGCCTCAGGAAGATCGCAC-3' |
| *priH* | priHfw | 5'-CTCTCGAGGCGGCCGCGCATCAGCATCTCTACACAC-3' |
|  | priHrv | 5'-GACTCGAGGCGGCCGCCCAGTCTATTACTTCTACGC-3' |
| *priI* | priIfw | 5'-CTCTCGAGGCGGCCGCGCGTAGAAGTAATAGACTGG-3' |
|  | priIrv | 5'-GACTCGAGGCGGCCGCCACTTTTCATCAAACTTATATTAATAAG-3' |
| *priJ* | priJfw | 5'-CTCTCGAGGCGGCCGCCTTATTAATATAAGTTTGATGAAAAGTG-3' |
|  | priJrv | 5'-GACTCGAGGCGGCCGCATATCTTACTTGCAGAGTGG-3' |
| *pAttb* | attbFW | 5'-AAATGGTGGGCATAATAGTGTTG-3' |
|  | attbRv | 5'-CCCAGACCGGCAACGAAAATCACG-3' |
| *pri* | oligo1pri | 5'-CTAAGCAAGTACGGCAGATATGTTCATAG-3' |
|  | WH3'/RB3' out | 5'-CCTCGATATACAGACCGATAAAAC-3' |

**Supplementary Table 2.** Sequence of the oligonucleotides used in this study.
